# Efficacy, safety and mechanism of Honghua Xiaoyao Pill in the treatment of peri-menopausal syndrome: A study protocol for a randomized controlled trial 

**DOI:** 10.3389/fphar.2022.1001228

**Published:** 2022-12-08

**Authors:** Xiao Wu, Lishan Zhou, Haoxu Dong, Man Tian, Shiqin Liu, Xiaohu Xu

**Affiliations:** ^1^ Department of Integrated Traditional Chinese and Western Medicine, Tongji Medical College, Tongji Hospital, Huazhong University of Science and Technology, Wuhan, China; ^2^ Department of Integrated Traditional Chinese and Western Medicine, Wuhan Children’s Hospital(Wuhan Maternal and Child Healthcare Hospital), Tongji Medical College, Huazhong University of Science and Technology, Wuhan, China; ^3^ Department of Integrated Traditional Chinese and Western Medicine, Maternal and Child Hospital of Hubei Province, Tongji Medical College, Huazhong University of Science and Technology, Wuhan, China

**Keywords:** Honghua Xiaoyao pill, randomized controlled trial, study protocol, peri-menopausal syndrome, Chinese patent medicine (CPM)

## Abstract

**Background:** Peri-menopausal syndrome (PMPS) has a high incidence rate and seriously affects the physical and mental health of women. Honghua Xiaoyao Pill (HHXYP) is a Chinese patent medicine, which has been reported to be used to treat PMPS. However, there is still a lack of randomized clinical trial to evaluate the efficacy and safety of HHXYP on life quality, mood and vasomotor symptoms for PMPS women. This study aims to investigate whether HHXYP is effective and safe in treating PMPS and the possible mechanism.

**Methods:** A multicenter, randomized, controlled clinical trial will be conducted in China to evaluate the efficacy and safety of HHXYP. Sixty women with peri-menopausal syndrome will be recruited at three centers and randomly in a 1:1 ratio to a treatment group using HHXYP (HHXYP group) and a control group using oryzanol (OC group). Participants will be treated with HHXYP or oryzanol for 12 weeks and followed up for 4 weeks. The primary outcome is the modified Kupperman Index (KI), which will be measured at baseline and 4, 8, 12, 16 weeks after randomization. The secondary outcomes include Hot flash scale (HFs), Menopause-Specific Quality of Life Scale (MENQOL) and Hamilton Depression/Anxiety Scale (HAMD/HAMA). The HFs are measured at the same point as the KI, other secondary outcomes are measured at baseline and 12, 16 weeks after randomization. The other outcomes are the levels of serum sex hormone, monoamine neurotransmitter, vascular vasomotor factor and the expression of phosphatidylinositol 3-active enzyme (PI3K)/protein activator enzyme B (Akt), which will be measured at baseline and 12 weeks after randomization. Adverse events will also be reported.

**Discussion:** HHXYP is a potential alternative Chinese patent medicine for PMPS. This trial will provide evidence for HHXYP on improving the quality of life, mood and vasomotor symptoms, and sex hormone levels of PMPS patients.

## 1 Introduction

Peri-menopause is defined as a period from 2 to 8 years before menopause and 1 year after final menstruation (Speroff, 2002). Nearly all women will experience this period, which is characterized by disturbing symptoms including hot flashes, night sweats, fatigue, insomnia, vaginal dryness and sexual dysfunction, clinically called “peri-menopausal syndrome”. These symptoms results from ovarian dysfunction, which leads to estrogen level fluctuations or reduction, hypothalamic-pituitary-ovarian axis dysfunction, and imbalance of neurotransmitters or hormones. Hot flashes and night sweats are the most frequently reported symptoms, which called as vasomotor symptoms (VMS). In addition, there is evidence to suggest that women during this reproductive transition were more likely to suffer from peri-menopause depression (PMD), leading to a reduced quality of life and increased burden for healthcare needs (Maki et al., 2019).

The conventional treatment for PMPS is hormone replacement therapy (HRT), but many patients refuse to long-term use this treatment because their concern for side-effects such as increased risks of endometrial and breast cancer, stroke, glucose intolerance and thromboembolic disease (Shapiro et al., 2013; Wassertheil-Smoller et al., 2003; Lumsden, 2016; Flores et al., 2021). Standard antidepressants have also been studied as a treatment for both the mood and vasomotor symptoms in peri-menopausal women, but many women are reluctant to use these because of their adverse effect such as weight gain, fatigue and drowsiness (Maki et al., 2018; Maki et al., 2019). Traditional Chinese medicine (TCM) has been widely used for more than 2,000 years to treat PMPS, especially the mood and vasomotor symptoms, with clinical therapeutic effects, safety and low cost.

Honghua Xiaoyao Pill (HHXYP), a Chinese patent medicine, modified from Xiaoyao san (pills), which is used to sooth liver-qi stagnation, activating blood and resolving stasis. It was composed of nine herbal medicines, including Angelica sinensis (Oliv.) Diels [Apiaceae; Angelicae Sinensis Radix], Atractylodes macrocephala Koidz [Asteraceae; Atractylodis Macrocephalae Rhizoma], Carthamus tinctorius L [Asteraceae; Carthami Flos], Paeonia lactiflora Pall [Paeoniaceae; Paeoniae Radix Alba], Poria cocos (Schw.) Wolf [Polyporaceae; Wolfiporia cocos (Schw.) Ryv.&Gibn], Mentha canadensis L [Lamiaceae; Menthae Haplocalycis Herba], Bupleurum chinense Wall. ex DC [Umbelliferae; Bupleuri Radix], Gleditsia sinensis Lam [Fabaceae; Spina Gleditsiae] and Glycyrrhiza uralensis Fisch. ex DC [Leguminosae; Glycyrrhizae Radix et Rhizoma]*.* Mi et al. established an analytical method combining UPLC-Q-TOF/MS and HPLC-QQQ/MS, preliminarily identified 55 components and quantified 14 prototype components of HHXYP (Mi et al., 2020). Another study revealed the key substances of HHXYP therapeutic effect on mammary gland hyperplasia by integrating metabolomics and network pharmacology analysis, including 7 targets, 6 herbs, and 17 ingredients (Gao et al., 2022). Previous study have reported that HHXYP combined with hormone replacement therapy can effectively increase serum estradiol level, reduce KI, MENQOL scores and relieve clinical symptoms of peri-menopausal syndrome (Zhang et al., 2015; Hai et al., 2021). Another study showed that HHXYP combined with Wenjing Decoction can effectively improve the sleep time of patients with peri-menopausal syndrome (Li et al., 2015). However, there is a lack of randomized clinical trials to evaluate the efficacy and safety of HHXYP on life quality, mood and vasomotor symptoms for PMPS women.

In this study, we will carry out a multicenter, randomized, controlled clinical trial to investigate whether HHXYP is effective and safe in treating PMPS. Furthermore, we will evaluate the impact and possible mechanism of HHXYP for life quality, mood and vasomotor symptoms.

## 2 Materials and methods

### 2.1 Study design

This is a multicenter, randomized, controlled trial to investigate the efficacy and safety of HHXYP by comparing with oryzanol. Sixty participants will be included from the following three hospitals: Tongji hospital, Tongji Medical College, Huazhong University of Science and Technology (HUST); Maternal and Child Hospital of Hubei Province, Tongji Medical College, HUST; Wuhan Maternal and Child Health Hospital, Tongji Medical College, HUST. Tongji Hospital is the central structure of the study.

**FIGURE 1:**
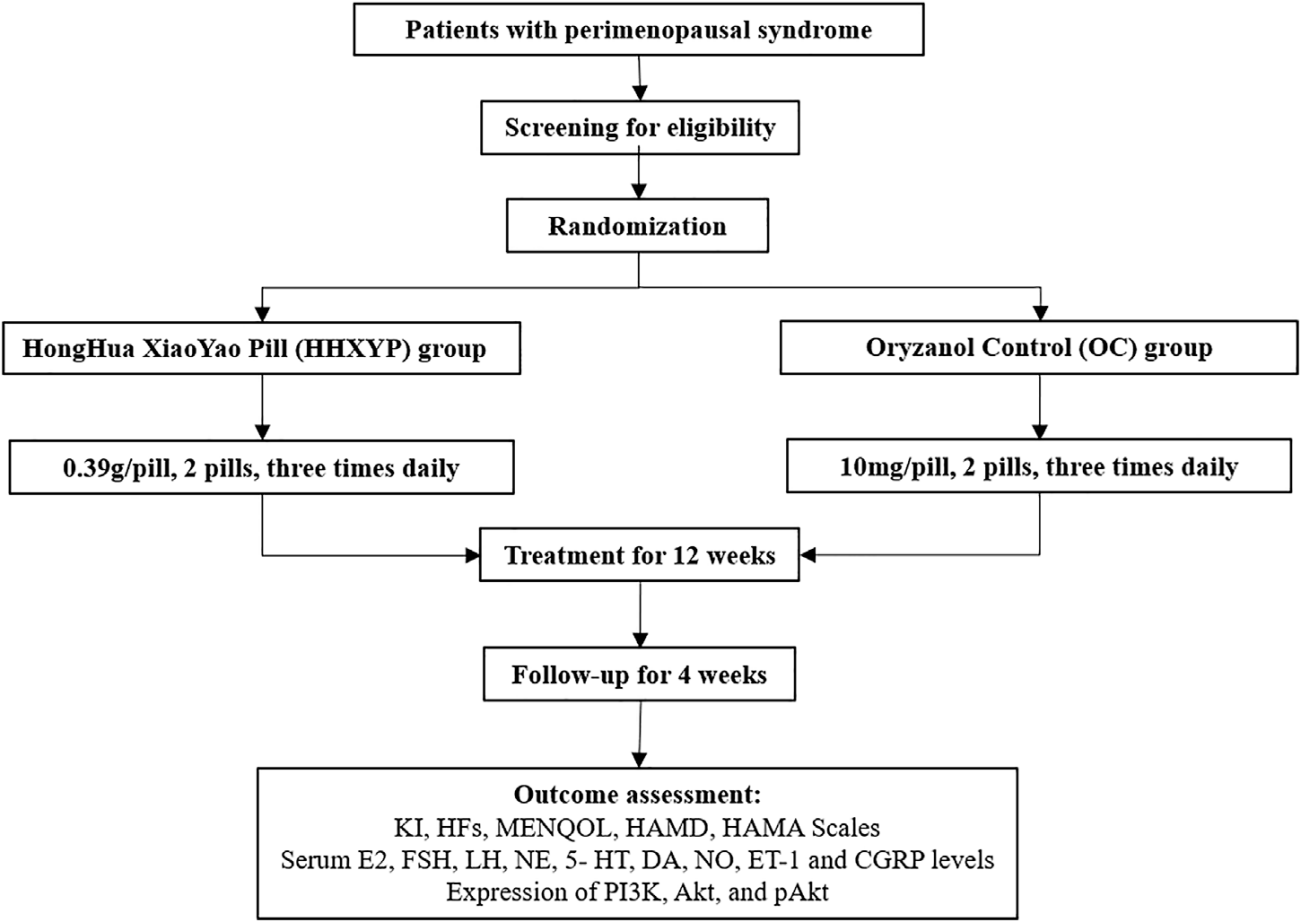
Flow diagram of the trial.

The duration of this study is 17 weeks including screening period of 1 week, treatment period of 12 weeks, and follow-up period of 4 weeks. Informed consent form signed by all qualified participants before enrollment. The Ethics Committee of Tongji Medical College, Huazhong University of Science and Technology approved this trial (approval number:2021S188). The protocol design conformed to the Declaration of Helsinki (Version Edinburgh 2000) and registered on the Chinese Clinical Trial Registry (ChiCTR2100050783). The flow chart of the study has been presented in Figure 1.

### 2.2 Randomization, allocation concealment and blinding

These participants will be randomly in a 1:1 ratio to HHXYP treatment group and OC group. An independent statistician will use SPSS software to generate the randomization sequence. The designated researcher in the central structure will prepare the assignments in opaque envelopes in sequence, and inform the researcher of the matching card number either by text message or *via* mobile communication application. To ensure blindness in this trial, both of HHXYP and oryzanol pills were packaged with the same appearance throughout the study process, outcome assessors and the data analysts will be blinded to the group allocation.

### 2.3 Participants

#### 2.3.1 Recruitment

This study focuses on women experiencing peri-menopausal syndrome. Participants will be recruited from hospitals and local community health service centers. Printed recruitment posters will be distributed in the outpatient of these hospitals and community health service centers. We will also use Internet, WeChat social software to briefly introduce our study to attract potential participants.

#### 2.3.2 Inclusion criteria

Participants are deemed peri-menopause according to STRAW classification (Harlow et al., 2012) and meet the following criteria:

1) Health women aged 45–60 years;
2) Menstrual disturbance (prolonged menstrual cycle or oligomenorrhea) lasting over 3 months, or amenorrhea for 2–12 months;
3) Suffering from vasomotor symptoms (hot flashes, sweating), somatic symptoms (insomnia, fatigue, headache, paresthesia), psychological symptoms (nervousness, melancholia), or urogenital symptoms (dyspareunia, vaginal dryness, urinary infection);
4) Serum follicle-stimulating hormone (FSH) > 10 IU/L, or decline of estradiol (E2);
5) Peri-menopausal symptoms have not been treated with HRT or other drugs in the last 2 months;
6) Uterus and bilateral accessories are complete and without resection;
7) Conscious and patients accepted informed consent.

#### 2.3.3 Exclusion criteria

Participants will be excluded from the trial if they meet any of the following criteria:

1) Did not meet the above Inclusion criteria;
2) Malignant ovarian tumors, ovaries or hysterectomy, premature ovarian failure, hysteromyoma ≥2 cm, and severe hyperplasia of mammary glands;
3) Acute gynecological infectious diseases or other acute infectious diseases;
4) Severe liver and kidney dysfunction, or other severe system diseases;
5) Use hormone drugs or Chinese medicine for treatment in the last 2 months;
6) have participated in other clinical trials;
7) Patients who failed to receive treatment as required or withdrew from the trial.

#### 2.3.4 Sample sizes

According to a previous study of peri-menopausal women in China (Zhang et al., 2014), we assumed that the mean ± standard deviation (SD) differences in the change in total KI score of the intervention group and the control group were 9 ± 7 and 3 ± 7. PASS 15 software was used for sample size calculation. We determined a sample size of 30 women for each group, with α equal to 0.05 (two-sided) and β equal to 0.01.

### 2.4 Interventions

#### 2.4.1 HongHua XiaoYao Pill group

Participants in HHXYP group will receive two HongHua XiaoYao pills (produced by Jiangxi Puzheng Pharmaceutical Co., Ltd. China, specification: 0.39g/pill) three times daily for 12 weeks.

#### 2.4.2 Oryzanol control group

Participants in OC group will receive two Oryzanol pills (produced by First Pharmaceutical Factory of Hainan Pharmaceutical Co., Ltd. China, specifications: 10mg/pill) three times daily for 12 weeks.

### 2.5 Outcomes

#### 2.5.1 Primary outcome

The primary outcome of the study is the modified Kupperman Index (KI), which will be measured at baseline and 4, 8, 12, 16 weeks after randomization. The total score of KI is 63. It includes 13 items related to peri-menopausal syndrome, and the severity score of each item ranges from 0 to 3. There were different basic score in each item: hot flashes and sweating = 4; Paresthesia, sleep disorders, nervousness, sexual complaints, urinary tract infection = 2; the other items = 1. Item score = basic score × severity score. Classification standard of disease condition: mild: symptom score <13; moderate: symptom score 14–26; severe: symptom score >27 points.

#### 2.5.2 Secondary outcomes

The secondary outcomes include the following items:

##### 2.5.2.1 Hot flash scale

The average frequency and severity of hot flash per week will be recorded by participants from baseline (-1 week) to week 16. The severity of hot flash was evaluated using visual analog scale (0–10). It also calculated baseline and 4, 8, 12, 16 weeks after randomization respectively.

##### 2.5.2.2 Menopause-specific quality of life scale

MENQOL is used to evaluate life quality of peri-menopausal women. It contains 27 questions related to vasomotor symptoms, psychological symptoms, physiological symptoms, and sexual life. A lower MENQOL score indicates PMPS has a less influence on life quality. It will be measured at baseline and 12, 16 weeks after randomization.

##### 2.5.2.3 Hamilton depression scale

HAMD will be used to evaluate the depressive condition of PMPS in this trial. Most of the items are scored on a 5-level scale with 0–4 points, a few items is a 3-level scale with 0–2 points. A higher score indicates the more severe depression. In this study, it will be evaluated at baseline and 12, 16 weeks after randomization.

##### 2.5.2.4 Hamilton anxiety scale

HAMA contains 14 items related to a number of anxiety symptoms. Each of items is scored on a 5-level scale with 0–4 points. A higher score indicates the more severe anxiety. In this study, it will be evaluated at baseline and 12, 16 weeks after randomization.

### 2.6 Mechanism outcomes

1) Sex hormone: the levels of E2, FSH and luteinizing hormone (LH) in serum are detected using ELISA.
2) Monoamine neurotransmitter: serum serotonin (5-HT), norepinephrine (NE) and dopamine (DA) levels were detected using ELISA.
3) Vascular vasomotor factor: serum nitric oxide (NO), calcitonin gene-related peptide (CGRP) and endothelin-1 (ET-1) levels were detected using ELISA.
4) Expression of serum protein and mRNA level of phosphatidylinositol 3-active enzyme (PI3K), protein activator enzyme B (Akt) and phosphorylated protein kinase B (pAkt) were detected using Western blot and polymerase chain reaction (PCR). These outcomes are measured before and after treatment.

### 2.7 Safety outcomes

In order to exclude serious heart, liver, and kidney diseases, all participants will also receive urine, stool, blood routine, liver function (ALT, AST), kidney function (BUN, creatinine) and ECG examination before and after treatment. Participants will inform the researchers adverse events or uncomfortable symptoms by WeChat software, telephone, or short message. Adverse events should be recorded in the Case Report Form and resolved immediately. Serious adverse events should be reported immediately to the principal investigator. The details of outcome assessment chart is given in Table 1.

**TABLE 1:** Trial processes chart.

Period	Enrollment	Baseline	Treatment			Follow-up
Time points	Week -1	Week 0	Week 4	Week 8	Week 12	Week 16
Patients						
Medical history	√					
Physical examination	√				√	
Laboratory examination	√				√	
Informed consent	√					
Randomization	√					
Intervention						
HongHua XiaoYao Pills (HHXYP) group			0.39g/pill, 2 pills, three times daily			
Oryzanol Control (OC) group			10mg/pill, 2 pills, three times daily			
Outcomes						
the modified Kupperman Index (KI)	√	√	√	√	√	√
Hot flash scale (HFs)	√	√	√	√	√	√
MENQOL		√			√	√
HAMD		√			√	√
HAMA		√			√	√
Serum E2, FSH and LH levels		√			√	
Serum NE, 5-HT and DA levels		√			√	
Serum NO, ET-1 and CGRP levels		√			√	
Expression of PI3K, Akt, and pAkt		√			√	
Trial evaluation						
Safety of medicine			√	√	√	√
Adverse event			√	√	√	√
Reasons of drop-outs or withdrawals			√	√	√	
Patient’s compliance					√	

E2, estradiol; FSH, follicle-stimulating hormone; LH, luteinizing hormone; NO, nitric oxide; ET-1, endothelin-1; CGRP, calcitonin gene-related peptide; NE, norepinephrine; 5-HT, serotonin; DA, dopamine; PI3K, phosphatidylinositol 3-active enzyme; Akt, protein activator enzyme B; pAkt, phosphorylated protein kinase B.

### 2.8 Statistical analysis

All data will be statistically analyzed using SPSS 20.0 software by a special analysts who is blinded to this trial. Continuous variables with normal distribution are presented as mean ± standard deviation (SD), and categorical variables are presented as frequency/percentage. All of the analyses will be performed on intention-to-treat (ITT) principle. Pearson’s chi-squared test or Fisher’s exact test are used to compare the frequency of categorical variables. Non-parametric Mann-Whitney test or Kruskal–Wallis test is used to compare data sets involving non-normal distribution. The comparison of continuous variables between groups will be evaluated by variance analysis. The main outcome scales at different time points will be analyzed by repeated measurement analysis of variance (ANOVA). Significance level is set as *p*-value less than 0.05.

### 2.9 Quality control

In order to guarantee the quality of trial, all investigators are asked to attend special training includes how to fill in the Case Report Form, how to record adverse reactions, how to teach the patients to take medicine, and other details of this trial.

### 2.10 Trial status

The participants are currently being recruited for the present study. Thirty-four patients have entered the screening period and nine patients have been enrolled until 1 June 2022.

## 3 Discussion

TCM has been widely used to treat PMPS for thousands of years in China (Fu et al., 2016). Recent years, it is increasingly popular abroad and thought to offer a personalized alternative to HRT (Johnson et al., 2019). The TCM believes that kidney deficiency is the foundation of peri-menopausal syndrome. As women enter the peri-menopausal period, the kidney’s reservation of Qi gradually decreases, resulting in decreased ovarian function and hormone levels, then causes VMS, such as hot flashes and night sweats. In addition, kidney deficiency also fails to nourish the viscera and meridians, leading to liver-qi stagnation and blood stasis. The function of the liver-qi mainly reflected in adjustment of spirit and emotion. Liver-qi stagnation and blood stasis are the important pathogenesis according to the theory of TCM. Its external manifestations are PMD include anxiety, depression, and other emotional disorders. Therefore, soothing and regulating liver-qi, activating blood and resolving stasis may be an important way to improve VMS and depression of peri-menopausal syndrome.

Honghua Xiaoyao Pill (HHXYP) is a Chinese patent medicine, which is used to sooth liver-qi stagnation, activating blood and resolving stasis. It is modified from Xiaoyao pills (wan), a prescription in Treatise on Febrile Diseases (*Shang Han Lun*). On the one hand, a study shows that Xiaoyao san played an anti-depression role by regulating the tryptophan-kynurenine metabolic pathways in depressed rats (Wang et al., 2018 ). Xiaoyao pills also has anti-inflammatory effect, which may relieve depression symptoms by inhibiting inflammatory reaction or activating NGF/BDNF-TRKA/TrkB-CREB pathway (Fang et al., 2020). On the other hand, Xiaoyao pills could reduce the level of inflammatory cytokines, enhance the expression of neurotrophic factors and synaptophysin to inhibite the behavior abnormality of LPS-induced depression-like model rats (Shi et al., 2019). Furthermore, Xiaoyao pills has antioxidant and neuroprotective effects on both cortical and hippocampal regions, which can improve depression-like behavior and oxidative stress of olfactory bulb excised rats by activating PIK3CA-AKT1-NFE2L2 BDNF signaling pathway (Ji et al., 2021). Therefore, it is reasonable to speculate that HHXYP has the effects of regulating anxiety and depression, anti-inflammation, and anti-oxidative stress. HHXYP has been reported as a complementary and alternative therapy for the treatment of gynecological diseases such as irregular menstruation, polycystic ovary syndrome and mastitis (Li 2014; Yan et al., 2020). There are also some researches indicated that it could alleviate peri-menopausal depression, insomnia and other peri-menopausal symptoms. HHXYP combined with estradiol tablets/estradiol dextroprogesterone tablets significantly reduced the modified KI, MENQOL score and serum FSH, LH levels, but increased serum E2 levels compare with estradiol tablets/estradiol dextroprogesterone tablets alone (Hai et al., 2021). HHXYP combined with Wenjing Decoction effectively improved the ovarian function and insomnia in patients with peri-menopausal syndrome compare with HHXYP alone (Zhang et al., 2015). HHXYP combined with conventional treatment (psychotherapy, vitamin D supplementation, HRT) improved Kupperman score and E2 levels compare with conventional treatment (Li et al., 2015). Based on the clues of clinical application and the theory of TCM, HHXYP seems show potential for treating PMPS. However, randomized controlled trials are still needed to determine. Therefore, this study will evaluate the efficacy of HHXYP for hot flash reduction, emotion improvement, and QOL improvement in PMPS patients. In order to better recruit participants, we set the age range between 45–60, as according to some reports, peri-menopause generally occurs around the age of 50 years, with a range between 40 and 60 years worldwide (Li et al., 2016).

In addition to using the KI, MENQOL, HAMD, and HAMA to evaluate the clinical efficacy, we will try to explore the possible mechanism of HHXYP treatment for PMPS by testing monoamine neurotransmitter and vascular vasomotor factor levels. As we known, vasomotor symptoms, such as hot flashes, is associated with increased risk of cardiovascular disease (Santoro, 2016). Serum nitric oxide (NO), a vascular vasomotor factor, plays a central role in the process of estrogen-induced cardioprotection. The increase of its concentration is associated with peri-menopausal status. The increase in plasma endothelin-1 (ET-1) is thought to reflect a rise in vascular production of the peptide in response to the impaired production of NO by endothelial cells (Beppu et al., 2002). Calcitonin gene-related peptide (CGRP) is another potent vascular vasomotor factor that plays an important role in maintaining vascular homeostasis. Both ET-1 and CGRP maintain balance in the body and regulate the vascular elasticity to maintain the systolic and diastolic function of local blood vessels. On the other hand, It is believed that changes in the levels of neurotransmitters, such as norepinephrine (NE), dopamine (DA), and serotonin (5-HT), are related to the occurrence of PMD. Studies have shown that the reduced of E2 level in women with PMD leads to a decrease in the expression of 5-HT, DA, and NE, while antidepressant effects can be achieved by adjusting the levels of 5-HT and DA (Schneider-Matyka et al., 2021). In recent years, it has been found that PI3K/AKT signaling pathway plays an important role the pathogenesis of peri-menopausal depression, which has been a close relationship with vascular factor (Cao et al., 2019; D'Antoni et al., 2017). We will try to explore the molecular mechanism of HHXYP in improving depression and hot flash in PMPS from PI3K/Akt signaling pathway.

Nevertheless, our study still has limitation. Firstly, placebo control is not set because of the potential harm of uncontrolled peri-menopausal syndrome. It might not be accurate enough to reflect the therapeutic effects of HHXYP. Secondly, our purpose is to clarify HHXYP therapeutic effect rather than a placebo effect, which that requires a positive drug as the control group. However, we didn’t use HRT as a drug control because the ethics committee considered that it might be carcinogenic to peri-menopausal women. Oryzanol is a kind of autonomic agent, which is widely used in the adjuvant treatment of insomnia, anxiety and depression in peri-menopausal women (Fujii et al., 2018). The mechanism study shows that oryzanol can increase the release of catecholamine related relatives in limbic system through the blood-brain barrier, which may be related to the improvement of stress (Fujii et al., 2018). In addition, some studies have found that oryzanol also has anti-inflammatory and antioxidant effects (Minatel et al., 2016; Ramazani et al., 2021). These effects are similar to the pharmacological effects of HHXYP, so we choose oryzanol, a recommended supplement in improving PMPS, as the control drug.

In conclusion, the results of this trial will help us to evaluate the efficacy, safety and possible mechanism of HHXYP in treating PMPS.

## Glossary

PMPS: Peri-menopausal syndrome
KI: the modified Kupperman Index
HFs: Hot flash scale
MENQOL: Menopause-Specific Quality of Life Scale
HAMD: Hamilton Depression Scale
HAMA: Hamilton Anxiety Scale Scale
VMS: vasomotor symptoms
PMD: perimenopause depression
HRT: hormone replacement therapy
HUST: Huazhong University of Science and Technology
FSH: follicle-stimulating hormone
E2: estradiol
SD: standard deviation
ITT: intention-to-treat analysis
LH: luteinizing hormone
NE: norepinephrine
UPLC-Q-TOF/MS: ultraperformance liquid chromatography-quadrupole time-of-flight/mass spectrometry
HPLC-QQQ/MS: high performance liquid chromatography-triple quadrupole mass spectrometry
5-HT: serotonin
DA: dopamine
NO: nitric oxide
ET-1: endothelin-1
CGRP: calcitonin gene-related peptide
PI3K: phosphatidylinositol 3-active enzyme
Akt: protein activator enzyme B
pAkt: phosphorylated protein kinase B
ELISA: enzyme-linked immunosorbent assay
PCR: polymerase chain reaction
ALT: alanine aminotransferase
AST: aspartate aminotransferase
BUN: blood urea nitrogen
ECG: electrocardiogram
TCM: Traditional Chinese medicine
QOL: Quality of Life

## References

[B1] Beppu M. Obayashi S. Aso T. Goto M. Azuma H. (2002). Endogenous nitric oxide synthase inhibitors in endothelial cells, endothelin-1 within the vessel wall, and intimal hyperplasia in perimenopausal human uterine arteries. J. Cardiovasc. Pharmacol. 39 (2), 192–200. 10.1097/00005344-200202000-00005 11791004

[B2] Cao L. H. Qiao J. Y. Huang H. Y. Fang X. Y. Zhang R. Miao M. S. (2019). PI3K-AKT signaling activation and icariin: The potential effects on the perimenopausal depression-like rat model. Mol. (Basel, Switz. 24 (20), 3700. 10.3390/molecules24203700 PMC6832648 31618892

[B3] D'Antoni S. Ranno E. Spatuzza M. Cavallaro S. Catania M. V. (2017). Endothelin-1 induces degeneration of cultured motor neurons through a mechanism mediated by nitric oxide and PI3K/akt pathway. Neurotox. Res. 32 (1), 58–70. 10.1007/s12640-017-9711-3 28285347

[B4] Fang Y. Shi B. Liu X. Luo J. Rao Z. Liu R. (2020). Xiaoyao pills attenuate inflammation and nerve injury induced by lipopolysaccharide in hippocampal neurons *in vitro* . Neural Plast. 2020, 8841332. 10.1155/2020/8841332 33014035 PMC7525321

[B5] Flores V. A. Pal L. Manson J. E. (2021). Hormone therapy in menopause: Concepts, controversies, and approach to treatment. Endocr. Rev. 42 (6), 720–752. 10.1210/endrev/bnab011 33858012

[B6] Fu S. F. Zhao Y. Q. Ren M. Zhang J. H. Wang Y. F. Han L. F. (2016). A randomized, double-blind, placebo-controlled trial of Chinese herbal medicine granules for the treatment of menopausal symptoms by stages. Menopause (New York, N.Y.) 23 (3), 311–323. 10.1097/GME.0000000000000534 26671188

[B7] Fujii M. Butler J. P. Sasaki H. (2018). Gamma-oryzanol for behavioural and psychological symptoms of dementia. Psychogeriatrics 18 (2), 151–152. 10.1111/psyg.12303 29417716

[B8] Gao Z. Mi R. Cheng Z. Li X. Zeng H. Wu G. (2022). Integrated metabolomics and network pharmacology revealed hong-hua-xiao-yao tablet's effect of mediating hormone synthesis in the treatment of mammary gland hyperplasia. Front. Pharmacol. 13, 788019. 10.3389/fphar.2022.788019 35177987 PMC8846323

[B9] Hai J. Li W. J. Wang W. X. Yang J. Dong X. C. (2021). Clinical study on the treatment of perimenopausal syndrome with the combination of Honghua Xiaoyao Tablets and estradiol tablets/estradiol diphosphate progesterone tablets composite packaging. Mod. Med. Clin. 36 (05), 981–985. Chinese.

[B10] Harlow S. D. Gass M. Hall J. E. Lobo R. Maki P. Rebar R. W. (2012). Executive summary of the stages of reproductive aging workshop + 10: Addressing the unfinished agenda of staging reproductive aging. Menopause (New York, N.Y.) 19 (4), 387–395. 10.1097/gme.0b013e31824d8f40 22343510 PMC3340903

[B11] Ji Y. Luo J. Zeng J. Fang Y. Liu R. Luan F. (2021). Xiaoyao pills ameliorate depression-like behaviors and oxidative stress induced by olfactory bulbectomy in rats via the activation of the PIK3CA-AKT1-nfe2l2/BDNF signaling pathway. Front. Pharmacol. 12, 643456. 10.3389/fphar.2021.643456 33935736 PMC8082504

[B12] Johnson A. Roberts L. Elkins G. (2019). Complementary and alternative medicine for menopause. J. Evid. Based. Integr. Med. 24, 2515690X19829380. 10.1177/2515690X19829380 PMC6419242 30868921

[B13] Li H. F. (2014). Effects of Honghua Xiaoyao Tablets on endocrine and metabolism in patients with polycystic ovary syndrome. China Women Child. Health Care 29 (22), 3622–3625. Chinese.

[B14] Li L. M. Zhou H. X. Yang Y. Zhu Q. Y. (2015). Application of Honghua Xiaoyao Tablets combined with Wenjing Decoction in improving ovarian function and insomnia during perimenopausal period. J. New Chin. Med. 47 (11), 127–128. Chinese. 10.13457/j.cnki.jncm.2015.11.058

[B15] Li R. X. Ma M. Xiao X. R. Xu Y. Chen X. Y. Li B. (2016). Perimenopausal syndrome and mood disorders in perimenopause: Prevalence, severity, relationships, and risk factors. Medicine 95 (32), e4466. 10.1097/MD.0000000000004466 27512863 PMC4985318

[B16] Lumsden M. A. (2016). The NICE guideline - menopause: Diagnosis and management. Climacteric 19 (5), 426–429. 10.1080/13697137.2016.1222483 27558301

[B17] Maki P. M. Kornstein S. G. Joffe H. Bromberger J. T. Freeman E. W. Athappilly G. (2019). Guidelines for the evaluation and treatment of perimenopausal depression: Summary and recommendations. J. Womens Health 28 (2), 117–134. 10.1089/jwh.2018.27099.mensocrec 30182804

[B18] Maki P. M. Kornstein S. G. Joffe H. Bromberger J. T. Freeman E. W. Athappilly G. (2018). Guidelines for the evaluation and treatment of perimenopausal depression: Summary and recommendations. Menopause (New York, N.Y.) 25 (10), 1069–1085. 10.1097/GME.0000000000001174 30179986

[B19] Mi R. Li X. Zhang Z. Cheng T. Tian S. Xu X. (2020). Chemical profiling of Honghua Xiaoyao tablet and simultaneous determination of its quality markers by liquid chromatography-tandem mass spectrometry combined with chemometrics methods. J. Sep. Sci. 43 (23), 4263–4280. 10.1002/jssc.202000689 32990401

[B20] Minatel I. O. Francisqueti F. V. Corrêa C. R. Lima G. P. (2016). Antioxidant activity of γ-oryzanol: A complex network of interactions. Int. J. Mol. Sci. 17 (8), 1107. 10.3390/ijms17081107 27517904 PMC5000585

[B21] Ramazani E. Akaberi M. Emami S. A. Tayarani-Najaran Z. (2021). Biological and pharmacological effects of gamma-oryzanol: An updated review of the molecular mechanisms. Curr. Pharm. Des. 27 (19), 2299–2316. 10.2174/1381612826666201102101428 33138751

[B22] Santoro N. (2016). Perimenopause: From research to practice. J. Womens Health 25 (4), 332–339. 10.1089/jwh.2015.5556 PMC4834516 26653408

[B23] Schneider-Matyka D. Grochans E. Lubkowska A. Panczyk M. Szkup M. (2021). The effect of tryptophan and serotonin levels on the severity of depressive and climacteric symptoms in perimenopausal women. Eur. Rev. Med. Pharmacol. Sci. 25 (9), 3425–3431. 10.26355/eurrev_202105_25822 34002815

[B24] Shapiro S. Farmer R. D. Stevenson J. C. Burger H. G. Mueck A. O. Gompel A. (2013). Does hormone replacement therapy (HRT) cause breast cancer? An application of causal principles to three studies. J. Fam. Plann. Reprod. Health Care 39 (2), 80–88. 10.1136/jfprhc-2012-100508 23493592

[B25] Shi B. Luo J. Fang Y. Liu X. Rao Z. Liu R. (2019). Xiaoyao pills prevent lipopolysaccharide-induced depression by inhibiting inflammation and protecting nerves. Front. Pharmacol. 10, 1324. 10.3389/fphar.2019.01324 31798446 PMC6863983

[B26] Speroff L. (2002). The perimenopause: Definitions, demography, and physiology. Obstet. Gynecol. Clin. North Am. 29 (3), 397–410. 10.1016/s0889-8545(02)00007-4 12353664

[B27] Wang J. Li X. He S. Hu L. Guo J. Huang X. (2018). Regulation of the kynurenine metabolism pathway by Xiaoyao San and the underlying effect in the hippocampus of the depressed rat. J. Ethnopharmacol. 214, 13–21. 10.1016/j.jep.2017.11.037 29217494

[B28] Wassertheil-Smoller S. Hendrix S. L. Limacher M. Heiss G. Kooperberg C. Baird A. (2003). Effect of estrogen plus progestin on stroke in postmenopausal women: The women's health initiative: A randomized trial. JAMA 289 (20), 2673–2684. 10.1001/jama.289.20.2673 12771114

[B29] Yan L. Y. Zhao H. M. Li C. Y. Liu H. Y. Kang Y. L. Fan W. (2020). Effects of Honghua Xiaoyao Tablets on blood WBC, CRP and PCT of patients with acute mastitis during lactation. Hebei Med. 42 (13), 2018–2021. Chinese.

[B30] Zhang J. Chen G. Lu W. Yan X. Zhu S. Dai Y. (2014). Effects of physical exercise on health-related quality of life and blood lipids in perimenopausal women: A randomized placebo-controlled trial. Menopause (New York, N.Y.) 21 (12), 1269–1276. 10.1097/GME.0000000000000264 24937024

[B31] Zhang L. Y. Cui Y. Gao C. X. (2015). Effects of Honghua Xiaoyao capsule on perimenopausal syndrome and serum sex hormones and endometrial thickness in patients. Med. Rev. 21 (21), 4024–4026. Chinese.

